# Bridging the Knowledge Gap: Enhancing RN Engagement in Antimicrobial Stewardship at MEDVAMC

**DOI:** 10.1017/ash.2025.263

**Published:** 2025-09-24

**Authors:** Alison Robins, Kihe Kim

**Affiliations:** 1 Baylor College of Medicine; 2 Michael E DeBakey Veterans Affairs Medical Center

## Abstract

**Background:** Antimicrobial resistance (AMR) poses a critical threat to global health, with healthcare-associated infections (HAIs) such as Clostridioides difficile and multidrug-resistant organisms (MDROs) exacerbated by antibiotic misuse. Up to 50% of inpatients receive antibiotics during their hospital stay, nearly 1/3 of which is inappropriate. The Standardized Antimicrobial Administration Ratio (SAAR) at Michael E DeBakey VA Medical Center (MEDVAMC) exceeded 1 from 2023–2024, signaling higher-than-expected antibiotic use. Nurses, as pivotal frontline healthcare providers, are often underutilized in antimicrobial stewardship program (ASP) efforts due to a lack of formal ASP education. Addressing this gap aligns with The Joint Commission standards, CDC guidelines, and ANA recommendations for improving ASP engagement and reducing HAIs. **Methods:** This quality improvement project utilized the Plan-Do-Study-Act (PDSA) framework to develop, implement, and refine an educational intervention aimed at enhancing RN knowledge and engagement in ASP. Baseline data, including a survey assessing RN ASP knowledge, informed the creation of a tailored training program. The program emphasized the 5D approach (Diagnosis, Drug, Dose, Duration, De-escalation), the role of nurses in ASP, and interdisciplinary collaboration. The initiative was endorsed by leadership and delivered through interactive workshops and case-based learning. Post-intervention surveys and infection rate analyses were conducted to evaluate outcomes. **Results:** The intervention led to a 92% increase in RN knowledge, with a mean post-intervention scores of 92 out of 100 among 67 participating nurses, compared to preintervention score of 48 out of 100. Improved RN competency in ASP facilitated stronger interdisciplinary communication and adherence to stewardship protocols, such as performing antibiotic time outs. Feedback from participants highlighted increased confidence in ASP roles and improved patient safety practices. Some examples of patient safety practices that improved, included more consistent documentation of allergy checks, antibiotic indications, and treatment plans within the electronic health record. Post-intervention, nurses felt more comfortable providing patient education on the importance of completing antibiotics, recognizing side effects, and infection prevention. **Conclusions:** Empowering nurses through targeted ASP education not only bridges critical knowledge gaps but also fosters a culture of safety and accountability in antibiotic use. Sustaining these outcomes requires integrating ASP education into routine RN training, continuous monitoring of infection rates, and leveraging interdisciplinary collaboration to maintain compliance with evidence-based stewardship practices. These findings underscore the transformative potential of nurse-led initiatives in combating AMR and improving healthcare outcomes.

